# The L motifs of two moss pentatricopeptide repeat proteins are involved in RNA editing but predominantly not in RNA recognition

**DOI:** 10.1371/journal.pone.0232366

**Published:** 2020-04-29

**Authors:** Takuya Matsuda, Mamoru Sugita, Mizuho Ichinose

**Affiliations:** 1 Center for Gene Research, Nagoya University, Chikusa-ku, Nagoya, Japan; 2 Institute of Transformative Bio-Molecules (WPI-ITbM), Nagoya University, Chikusa-ku, Nagoya, Japan; University of Cyprus, CYPRUS

## Abstract

Pentatricopeptide repeat (PPR) proteins, composed of PPR motifs repeated in tandem, are sequence-specific RNA binding proteins. Recent bioinformatic studies have shown that the combination of polar amino acids at positions 5 and last in each PPR motif recognizes RNA bases, and an RNA recognition code for PPR proteins has been proposed. Subsequent studies confirmed that the P (canonical length) and S (short) motifs bind to specific nucleotides according to this code. However, the contribution of L (long) motifs to RNA recognition is mostly controversial, owing to the presence of a nonpolar amino acid at position 5. The PLS-class PPR protein PpPPR_56 is a mitochondrial RNA editing factor in the moss *Physcomitrella patens*. Here, we performed *in vitro* RNA binding and *in vivo* complementation assays with PpPPR_56 and its variants containing mutated L motifs to investigate their contributions to RNA recognition. *In vitro* RNA binding assay showed that the original combination of amino acids at positions 5 and last in the L motifs of PpPPR_56 is not required for RNA recognition. In addition, an *in vivo* complementation assay with RNA editing factors PpPPR_56 and PpPPR_78 revealed the importance of nonpolar amino acids at position 5 of C-terminal L motifs for efficient RNA editing. Our findings suggest that L motifs function as non-binding spacers, not as RNA-binding motifs, to facilitate the formation of a complex between PLS-class PPR protein and RNA. As a result, the DYW domain, a putative catalytic deaminase responsible for C-to-U RNA editing, is correctly placed in proximity to C, which is to be edited.

## Introduction

Pentatricopeptide repeat (PPR) proteins are found in all eukaryotes and constitute one of the largest protein families in terrestrial plants, including over 400 members in flowering plants [1, 2]. In plant organelles, PPR proteins bind to specific target RNAs and participate in various RNA processing events, including RNA stabilization, splicing, and editing [3, 4]. PPR proteins are members of the α-solenoid superfamily of helical repeat proteins and grouped into P- and PLS-classes. P-class proteins have only the canonical 35 amino acid PPR motif (P motif), whereas PLS-class proteins generally comprise arrays of PLS triplets (L, long variant of P motif; S, short variant of P motif). Further, the consensus sequence profile of the last PLS triplet, named as P2-L2-S2, is different from that of the canonical PLS triplet [5]. To date, many PLS-class proteins have been reported to be involved in RNA editing, which converts specific cytidines (C) to uridines (U) in organellar transcripts [6–8]. The PLS proteins present additional C-terminal domains, termed as the extension (E) and DYW domains. The E domain comprises two PPR-like motifs (each composed of 34 amino acids) [5] and could be involved in a sequence-specific interaction with its RNA ligand as it has been shown for CRR2 [9]. The cytidine deaminase DYW domain is named for its conserved last three amino acids, Asp-Tyr-Trp [2], and exhibits the cytidine deaminase activity *in vitro* [10]. These PLS-class proteins bind to target RNAs in a sequence-specific manner in proximity to the C to be edited [10, 11].

Recent studies have provided an insight into how PPR proteins bind to specific target RNAs. Bioinformatic analyses have revealed the combinations of amino acids at positions 5 and last in each PPR motif that recognize RNA bases [12–14]. The proposed RNA recognition code for PPR proteins (hereafter referred to as the PPR code) was confirmed from the recoding of the native PPR proteins and changing their specificities *in vitro* and *in vivo*. The P and S motifs bind to specific nucleotides based on the PPR code [12, 15, 16]. However, the contribution of the L motif to RNA recognition remains controversial. Barkan *et al*. [12] suggested that the L motif shows no binding activity to RNA bases because the amino acid profile at position 5 of the L motifs is markedly different from the profiles for P and S motifs. In addition, an *in vitro* RNA binding assay using the *Arabidopsis thaliana* RNA editing factor CLB19 and its variant proteins showed that the L motif does not participate in base-specific RNA recognition [16]. Computational analyses in other studies, however, have proposed a PPR code for the L motif [13, 14].

Here, we systematically mutated the L motifs of the *Physcomitrella patens* RNA editing factor PpPPR_56 and performed an *in vitro* RNA binding assay to investigate their contribution to RNA recognition. We also analyzed the functions of the L motifs in RNA editing by an *in vivo* complementation assay using variants of the editing factors PpPPR_56 and PpPPR_78. We found that the original combinations of amino acids at positions 5 and last in the L motifs of PpPPR_56 were deemed less important for RNA recognition. Nevertheless, the nonpolar amino acids at position 5 of the C-terminal L motifs were necessary for RNA editing.

## Materials and methods

### Plant material and growth conditions

The moss *P*. *patens* was grown on BCDATG plates at 25°C under continuous light [17]. The *PpPPR_56* knockout (KO) line (Δ*56*–10) and *PpPPR_78* KO line (Δ*78*–19) were prepared as previously described [18, 19].

### Protein expression and purification

The region encoding PpPPR_56 (amino acids 161–764) without the N-terminal transit peptide and the DYW domain was amplified from the plasmids prepared for *in vivo* analysis. The amplicons were cloned into the pBAD/Thio-TOPO vector (Invitrogen) in-frame with thioredoxin (Trx) at the N-terminus and 6× histidine (His)-tag at the C-terminus. All recombinant proteins were expressed in the *Escherichia coli* BL21 strain (Novagen). The cells were grown at 37°C in Luria Bertani (LB) medium supplemented with 50 μg mL^−1^ ampicillin until the OD600 reached 0.4 and then cooled down at 4°C for 30 min. Protein expression was induced with the addition 0.2% L-arabinose and cells were kept shaking for 18 h at 16°C, followed by centrifugation at 5,000 ×*g* at 4°C for 10 min. The obtained pellets were resuspended in the lysis buffer included in the EzBactYeast Crusher kit (ATTO) along with protease inhibitor and DNase I, according to the manufacturer’s instructions. The supernatant was collected following centrifugation at 10,000 ×*g* at 4°C for 5 min and incubated with 1/100 volume of Ni-NTA agarose beads (Qiagen) at 4°C for 1 h with shaking. The mixture was loaded on a column and washed thrice with 5 mL of a washing buffer (50 mM Tris, pH 8.0, 500 mM potassium chloride, 10 mM magnesium chloride, 0.5% Triton X-100, 10% glycerol, 1 mM dithiothreitol) containing 20 mM imidazole. The recombinant protein was eluted with 150 μl of an elution buffer (washing buffer containing 250 mM imidazole) and then dialyzed using a dialysis buffer (20 mM Tris, pH 8.0, 150 mM sodium chloride, 10% glycerol) overnight at 4°C.

### RNA Electrophoresis Mobility Shift Assay (REMSA)

The synthetic oligo RNAs, nad3-edit (5′-UUAUUAUAUUUGAUUUGGAAGUCACCUUUU**C**AUUUC-3′), nad4-edit (5′-AUUUUUAUAUAGGUAUAGACGGUAUCUCUU**C**AUUUU-3′), and PpPsbI-RNA1 (5′-UUAUUUUUUUCGUUUCUCUUUUUGUUUUU-3′) [20], were used for REMSA (editing sites are underlined). Each synthetic oligo RNA was labeled at its 5′-end using T4 polynucleotide kinase (TaKaRa) and [γ-^32^P] ATP at 37°C for 1 h, and then extracted by ethanol precipitation. REMSA was performed according to the method of Goto *et al*. [21] with a few modifications. The recombinant protein (concentration range from 0 to 100 nM) was incubated at 25°C for 10 min in a reaction mixture comprising 40 mM Tris, pH 8.0, 100 mM sodium chloride, 4 mM dithiothreitol, 0.1 mg mL^−1^ bovine serum albumin (BSA), and 10% glycerol. A ^32^P-labeled synthetic RNA probe (50 pM) was added to the mixture, and then incubated for 15 min. The reaction mixture was subjected to native 6% polyacrylamide gel electrophoresis. Free- and protein-bound RNAs in the gel were imaged with a STORM 820 Phosphoimager (GE Healthcare). The fraction of bound oligonucleotides was quantified with ImageQuant (GE Healthcare). The binding curves were plotted using Prism (GraphPad software).

### Construction of PpPPR_56 and PpPPR_78 variants for *in vivo* complementation assay and moss transformation

The primers used in this study are listed in S1 Table. The full-length *PpPPR_56* cDNA coding region containing the native stop codon was cloned into the *Swa*I site of p9WmycH13 overexpression vector [22]. A sequence encoding a 3× HA epitope tag with a triplet amino acid V-Y-K linker at the C-terminus was derived from pPHG-HA3 [23] and amplified with its respective primers to append a 15-nucleotide cloning adaptor via the In-Fusion HD cloning system (Clontech). The fragment product was inserted between the transit peptide and the first PPR motif (residues 140 and 141) of PpPPR_56, and the obtained plasmid was termed as p56wtHA. The region of the genomic DNA corresponding to the full-length *PpPPR_78* gene, which includes the 164 bp upstream and the native stop codon, was amplified from genomic DNA and cloned into the *Swa*I site of p9WmycZ3 overexpression vector [21]. The resultant plasmid was named as p78wt. The *PpPPR_56* and *PpPPR_78* variants were amplified from p56wtHA and p78wt, respectively, using mutagenic primers including mutated bases and PrimeSTAR GXL DNA polymerase (TaKaRa). The constructs were sequenced to confirm the mutations, and then linearized and introduced into the *PpPPR_56* KO (Δ*56*–10) or *PpPPR_78* KO (Δ*78*–19) lines either by particle bombardment or poly ethylene glycol-mediated DNA transformation [17, 24]. Transgenic lines were selected using of 50 μg mL^−1^ hygromycin or 100 μg mL^−1^ zeocin in BCDAT medium. Transgene expression in complemented mosses was confirmed by reverse transcription-polymerase chain reaction (RT-PCR) (see RNA editing analysis section) with appropriate primers (S1 Table) and PrimeSTAR Max DNA polymerase (TaKaRa).

### RNA editing analysis

DNA-free RNA was extracted from 4-day-old protonemata using ISOGEN II (Nippon Gene Co., Ltd.), and 1 μg DNase-treated RNA was subjected to cDNA synthesis using ReverTra Ace (TOYOBO) and a random hexamer primer. The editing sites were amplified using SapphireAmp^®^ Fast PCR Master Mix (TaKaRa) and gene-specific primers listed in S1 Table. PCR products were treated with ExoSAP-IT Express PCR Cleanup Regent (Thermo Fisher Scientific) to remove free primers and dNTPs, and subjected to sequencing reactions using forward primers and BigDye Terminator v3.1 (Thermo Fisher Scientific). The chromatographs were analyzed using DNADynamo (Blue Tractor Software Ltd.) to quantify the editing efficiency corresponding to the ratio of the height of T and the sum of the heights of C and T. The RNA editing efficiency values with error bars corresponded to the mean of three replicates.

## Results

### PpPPR_56 specifically binds to target RNAs

**Fig 1:**
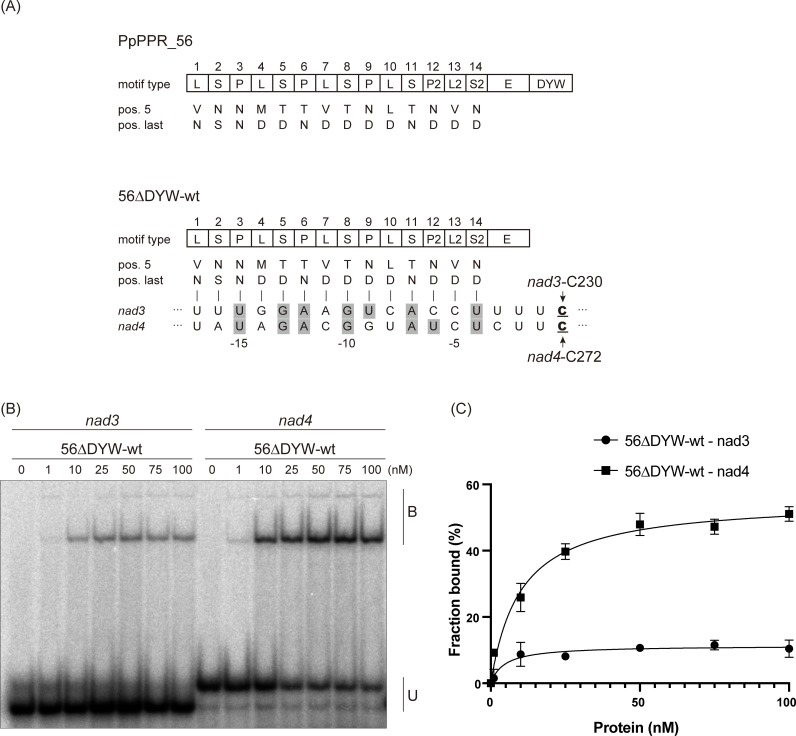
PpPPR_56 alignment and binding to the *nad3* and *nad4* target sites. (A) Alignment of PpPPR_56 and its RNA targets *nad3* and *nad4*. The key amino acids at positions 5 and last of each PPR motif are indicated. The editing sites C are underlined. The aligned nucleotides are shaded in gray to indicate matches to RNA recognition code, as proposed by Barkan *et al*. [12]. (B) REMSA was performed using recombinant 56ΔDYW-wt protein and 50 pM of ^32^P-labeled RNAs (*nad3* and *nad4*). 56ΔDYW-wt concentrations ranging from 0 to 100 nM are shown above each lane. B and U indicate the bound and unbound probe, respectively. (C) Binding was quantitated from the comparison between the bound (B) and the unbound (U) probes. Each reaction was performed in triplicates. Error bars indicate ± standard deviation (SD).

We have previously reported that the *P*. *patens* mitochondrial PPR-DYW protein, PpPPR_56, is an RNA editing factor for *nad3*-C230 and *nad4*-C272 sites [18, 25]. Here, we adopted the PPR code of Barkan *et al*. [12]. The last PPR motif of PLS-class PPR proteins is aligned with the fourth nucleotide upstream of the target C [12, 14]. According to the proposed PPR code, the alignment of PpPPR_56 to both RNA targets revealed seven matches and seven gaps among 14 PPR motifs (Fig 1A). To confirm whether PpPPR_56 is a site-recognition factor, a variant of PpPPR_56 lacking the transit peptide and the C-terminal DYW domain was expressed in *E*. *coli* in the form of a fusion protein to thioredoxin (Trx) and 6× His tag at the N- and C-terminus, respectively (56ΔDYW-wt; S1 Fig). Recombinant protein PpPPR_56 without the DYW domain was used, as we were unable to purify PpPPR_56 carrying the DYW domain from the soluble fraction. REMSA was performed using the target RNAs *nad3* and *nad4* (Fig 1) and the non-target RNA *psbI* (S1 Fig). The 56ΔDYW-wt could bind to its natural targets (*nad3* and *nad4*) but had a better affinity toward *nad4* than toward *nad3* (Fig 1B and 1C). In contrast, it did not bind to the non-target *psbI* (S1 Fig). This binding preference between the 56ΔDYW-wt and its target RNAs reflects the editing efficiency observed in previous work in *P*. *patens* and bacteria (~80% for *nad3*-C230 and ~100% for *nad4*-C272 [18, 25, 26]).

### Altered L motifs do not affect PpPPR_56 binding affinity

**Fig 2:**
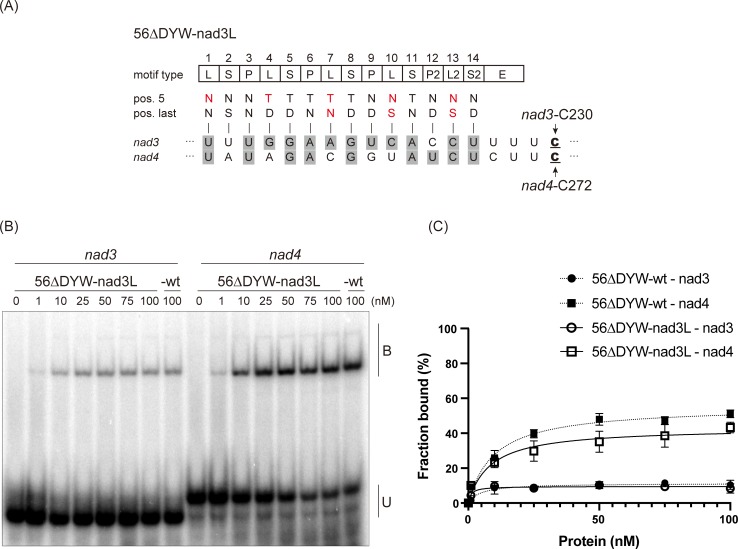
*In vitro* RNA binding assay for the PpPPR_56 variant. (A) Alignment of the 56ΔDYW-nad3L variant to *nad3* and *nad4* RNAs. The key amino acids at positions 5 and last of each PPR motif are indicated. Altered amino acid residues are shown in red. The editing sites C are underlined. The aligned nucleotides are shaded in gray to indicate the matches to the proposed RNA recognition codes. (B) REMSA was performed using recombinant 56ΔDYW-nad3L, 56ΔDYW-wt, and ^32^P-labeled RNAs (*nad3* and *nad4*). The recombinant protein concentrations are shown above each lane. B and U indicate the bound and unbound probe, respectively. (C) Binding curves representing the fraction of the bound probe indicated in Figs 1B and 2B. Error bars indicate ± SD (*n* = 3).

PpPPR_56 has five L motifs, none of which features amino acids at positions 5 and last characteristic of the established PPR code. To investigate the contribution of the L motifs to RNA recognition, we created a 56ΔDYW-nad3L variant by mutating all the L motifs of PpPPR_56 to allow recognition of nucleotides in the *nad3*-C230 *cis*-sequence. For instance, the first PPR motif of PpPPR_56 is the L motif (hereafter, 1L) containing a valine and an asparagine at positions 5 and last, respectively (Fig 1A). 1L was aligned to U at position −17 from *nad3* and *nad4* editing sites. At 1L, the variant 56ΔDYW-nad3L contained the [NN] combination, which should recognize C or U, instead of [VN]. Similar to 1L, the 4L[MD], 7L[VD], 10L[LD], and 13L[VD] were converted to 4L[TD], 7L[TN], 10L[NS], and 13L[NS], respectively, in 56ΔDYW-nad3L (Fig 2A). As a result, 56ΔDYW-nad3L had 12 matches and two gaps with respect to the *nad3* sequence and nine matches and five gaps with respect to the *nad4* sequence (Fig 2A). We expressed 56ΔDYW-nad3L and performed REMSA using *nad3* and *nad4* probes. In comparison with 56ΔDYW-wt, this variant showed a slight decrease in binding toward the *nad4* probe (Fig 2B and 2C), instead of no significant difference observed for its binding preference to *nad3* probe, although five L motifs were modified to recognize the *nad3* sequence. This result suggests that the L motifs of PpPPR_56 make only little contribution to RNA binding.

### The 56nad3L variant showed no recovery in RNA editing ability

**Fig 3:**
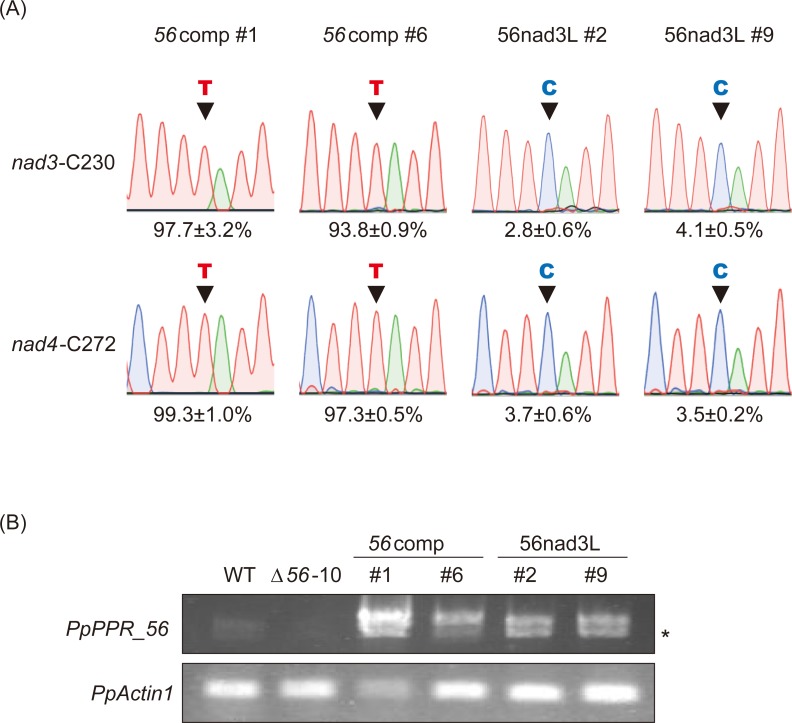
*In vivo* complementation assay with the PpPPR_56 variant. (A) RNA editing in *PpPPR_56* KO mosses complemented with PpPPR_56 (*56*comp) and 56nad3L. The cDNA sequencing chromatograms are shown. Black arrowheads indicate editing sites. The average editing efficiency is shown as percentage (*n* = 3). (B) RT-PCR analysis of *PpPPR_56* transcript in the wild-type (WT), *PpPPR_56* KO line (Δ*56*–10), and Δ*56* complemented mosses. *PpActin1* was used as a control. The asterisk indicates an alternative splicing variant of *PpPPR_56*.

To investigate whether the altered L motifs affect RNA editing, we introduced the wild-type *PpPPR_56* and its *56nad3L* variant into *PpPPR_56* KO moss line (Δ*56*–10 [18]) and selected at least two independent stable complemented lines per construct (e.g., *56*comp lines #1 and #6; Fig 3). A 3× HA epitope tag sequence was inserted in front of the first PPR motif to not affect the editing function of the C-terminal DYW domain [27]. As we were unable to detect any bands by immunoblot using anti-HA-tag antibody, we verified the expression of the transgene in the complemented Δ*56*–10 lines by RT-PCR. The wild-type PpPPR_56 (*56*comp) completely rescued the RNA editing defects of *nad3*-C230 and *nad4*-C272 in the *PpPPR_56* disruptant (Fig 3A), indicating that the HA-tag had no effect on the function of PpPPR_56. The *56nad3L* variant unexpectedly did not complement the *PpPPR_56* KO moss (Fig 3), although the recombinant 56ΔDYW-nad3L protein could bind to both *nad3* and *nad4* probes (Fig 2B). This result shows that the alteration in the amino acids at positions 5 and last of the L motifs in PpPPR_56 impaired its RNA editing function.

### The C-terminal L motifs in PpPPR_56 are essential for RNA editing

**Fig 4:**
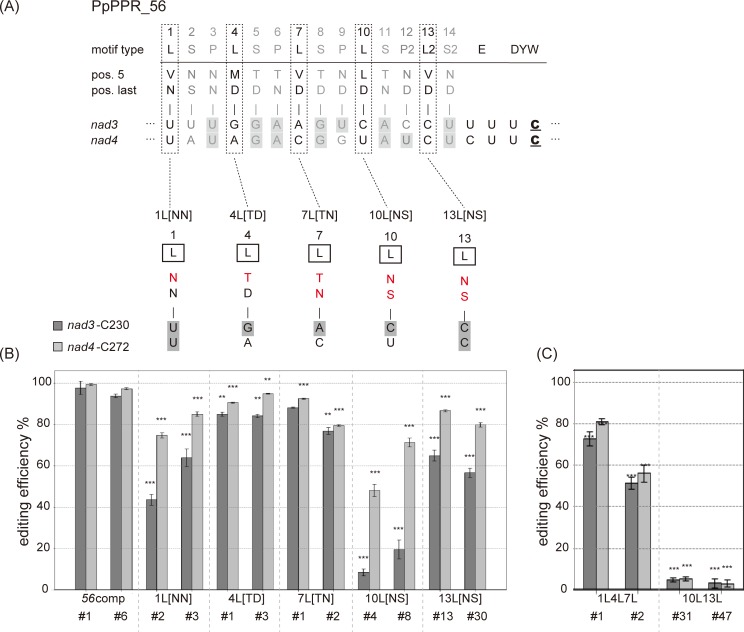
Effect of mutations of individual PPR motifs in PpPPR_56 *in vivo*. (A) Alignment of PpPPR_56 and its variants to the *nad3* and *nad4* RNAs. Key amino acids at positions 5 and last of each PPR motif are indicated. Altered amino acid residues are shown in red. Aligned nucleotides are shaded in gray to indicate matches to the proposed RNA recognition codes. RNA editing levels in *PpPPR_56* KO mosses complemented with the variants for which the individual motifs were altered (B), as well as with 1L4L7L and 10L13L variants for which multiple motifs were modified (C). Error bars indicate ± SD (*n* = 3). Significant differences (Student’s *t*-test) are shown by asterisks: *P < 0.05, **P < 0.01, and ***P < 0.001.

To investigate which alterations of PPR codes in PpPPR_56 affected the editing function, we mutated its five individual L motifs (1L, 4L, 7L, 10L, and 13L) and transformed the mutated PpPPR_56 into Δ*56*–10. The 1L[NN] variant has the first PPR motif with [NN] instead of [VN] (Fig 4A), while the [NN] combination was expected to recognize the pyrimidine base, C or U. This variant showed lower editing efficiency at the *nad3* and *nad4* sites than *56*comp (Fig 4B). Further, a significant decrease in editing efficiency, especially at the *nad3* site, was observed for the 10L[NS] and 13L[NS] variants. On the other hand, the editing efficiency almost recovered for the 4L[TD] and 7L[TN] variants to the level reported for the *56*comp line (Fig 4B).

To identify the components of the L motifs that are important for RNA editing, we created the variants 1L4L7L and 10L13L. Three N-terminal L motifs were modified to recognize the *nad3* sequence in 1L4L7L, and two C-terminal L motifs were modified in 10L13L (Fig 4C). As shown in Fig 4, the 1L4L7L variant showed partial recovery of RNA editing function, like as 1L[NN] transgenic lines. In contrast, the RNA editing efficiency at the *nad3* and *nad4* sites was less than 5% in the 10L13L variant (Fig 4C). It is surprising that *nad4* editing was severely impaired for the 10L13L variant because more than 50% of the editing occurred at the *nad4* site in 10L[NS] and 13L[NS] variants (Fig 4B). These results indicate that the C-terminal L motifs in PpPPR_56 are more critical for RNA editing than the N-terminal ones.

### Nonpolar amino acids at position 5 of the C-terminal L motifs are required for efficient RNA editing

The 10L[NS] and 13L[NS] variants exhibited altered amino acids at positions 5 and last in 10L[LD] or 13L[VD] (Fig 4B). To determine the position that influences RNA editing, we mutated either of the amino acids at position 5 or last of the C-terminal L motifs (S2 Fig). RNA editing efficiency was impaired in the 10L[ND] and 13L[ND] variants but fully recovered for the 10[LS] and 13L[VS] variants (S2 Fig). These results suggest that the amino acids at position 5 are more important for RNA editing than those at position last in the C-terminal L motifs.

**Fig 5:**
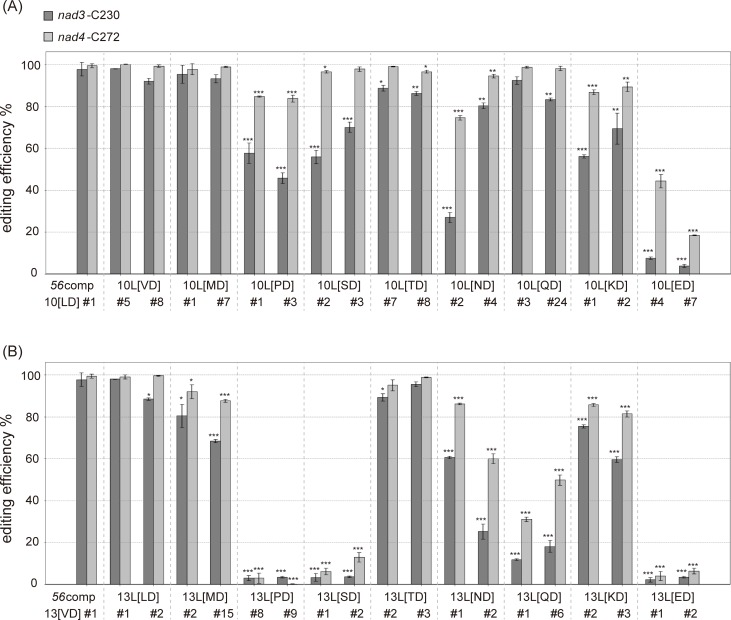
Effect of mutation of the C-terminal PPR motifs of PpPPR_56 *in vivo*. RNA editing levels in *PpPPR_56* KO mosses complemented with the variants. The amino acid combination of the tenth (A) or thirteenth (B) PPR motif was replaced by each combination shown. Error bars indicate ± SD (*n* = 3). Significant differences (Student’s *t*-test) are shown by asterisks: *P < 0.05, **P < 0.01, and ***P < 0.001.

We then investigated the types of amino acids at position 5 of the C-terminal L motifs that are required for efficient RNA editing. We systematically substituted the amino acids at position 5 of 10L or 13L with other amino acids, and introduced these variants into Δ*56*–10. The 10L[VD] variant had nonpolar valine instead of a leucine at position 5 of the tenth PPR motif. As a result, the editing efficiency of the 10L[VD] variant was fully recovered (Fig 5A). The 10L[MD] variant also showed complete recovery of editing efficiency. In contrast, a significant decrease in RNA editing efficiency was observed for the 10L[PD] variant. The variants containing polar amino acids such as serine (S), asparagine (N), lysine (K), and glutamate (E) at position 5 showed impaired editing efficiency at *nad3* and/or *nad4* (Fig 5A).

The 13L[LD] variant had a leucine at position 5 of its 13th PPR motif, and its editing efficiency was restored to the level of the wild-type (Fig 5B). However, the editing efficiency of the 13L[MD] variant failed to completely recover. RNA editing in the 13L[PD] variant was significantly impaired, consistent with the observation for the 10L[PD] variant (Fig 5). Proline cannot form hydrogen bonds with other amino acids, and its side-chain sterically hinders the formation of α-helix [28]. Variants with polar amino acids at position 5 showed impaired RNA editing, and replacement of valine (V) at position 5 with serine (S), asparagine (N), glutamine (Q), or glutamate (E) severely impaired RNA editing at *nad3* and *nad4* (Fig 5B). We investigated whether structural changes in the PPR-RNA complex caused by proline or polar amino acids at position 5 in 10L or 13L leads to inefficient RNA editing. We expressed 56ΔDYW-13L[PD] and 56ΔDYW-13L[ED] (whose 13L[VD] is mutated into 13L[PD] or 13L[ED]) (S1 Fig) and performed REMSA. Both proteins could bind to the *nad3* and *nad4* RNA probes, like as 56ΔDYW-wt. Thus, the low editing efficiency of 13L[PD] or 13L[ED] was not attributed to the inhibition of the PPR-RNA complex (S3 Fig). In conclusion, the L motifs of PpPPR_56 predominantly participate in RNA editing, but not RNA binding, and the nonpolar amino acids at position 5 of the C-terminal L motifs are especially important for efficient RNA editing.

### The two C-terminal L motifs in PpPPR_78 are important for efficient RNA editing

**Fig 6:**
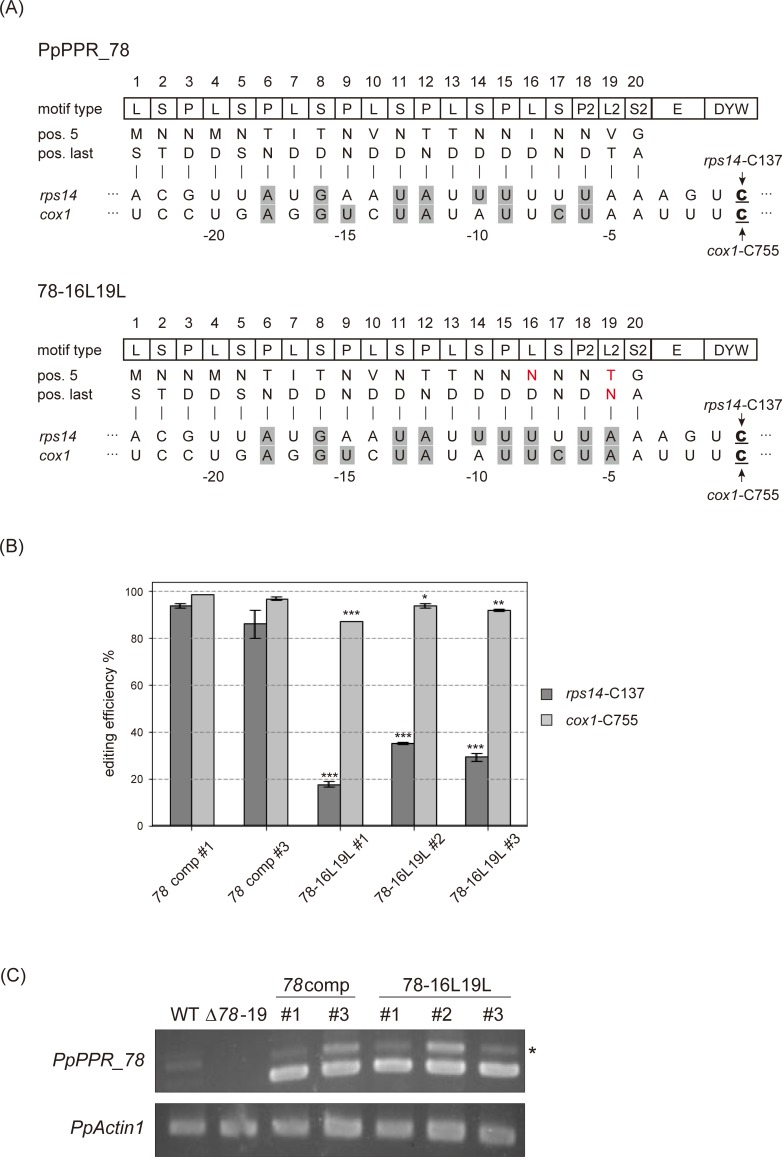
*In vivo* complementation assay with the PpPPR_78 variant. (A) Alignment of PpPPR_78 and 78-16L19L variant to their target RNAs, *rps14* and *cox1*, respectively. The key amino acids at positions 5 and last of each PPR motif are indicated. The altered amino acid residues are shown in red. The editing sites C are underlined. The aligned nucleotides are shaded in gray to indicate matches to the proposed RNA recognition codes. (B) RNA editing levels in *PpPPR_78* KO mosses complemented with PpPPR_78 (*78*comp) and 78-16L19L. Error bars indicate ± SD (*n* = 3). Significant differences (Student’s *t*-test) are shown by asterisks: *P < 0.05, **P < 0.01, and ***P < 0.001. (C) RT-PCR for *PpPPR_78* transcript in wild-type (WT), *PpPPR_78* KO mutant (Δ*78*–19), and Δ*78* complemented lines (*78*comp and 78-16L19L). *PpActin1* was used as a control. The asterisk indicates *PpPPR_78* pre-mRNA with the second intron.

To determine whether the PpPPR_56 results could be extended to another editing factor, we analyzed the PLS-class PpPPR_78 that has 20 PPR motifs and is involved in RNA editing at the *rps14*-C137 and *cox1*-C755 sites in mitochondria [19, 29]. We created the 78-16L19L variant, wherein 16L[ID] and 19L[VT] were changed to 16L[ND] and 19L[TN] for the recognition of U and A, respectively. We introduced each of them into the *PpPPR_78* KO mutant (Δ*78*–19 [19]) (Fig 6). The 78-16L19L variant showed a marked decrease in *rps14* editing efficiency and a minor reduction in *cox1* editing efficiency as compared with the wild-type PpPPR_78 (*78*comp) (Fig 6B). Therefore, the C-terminal L motifs of PpPPR_78 are critical for RNA editing like as PpPPR_56.

### Discussion

The contribution of the P and S motifs to RNA recognition in PLS-class PPR proteins has been experimentally confirmed [15, 16]. However, whether L motifs also recognize RNA bases is yet unclear. The P and S motifs usually have polar amino acids such as asparagine, threonine, and serine at position 5 [12–14] that recognize their corresponding ribonucleotides by forming hydrogen bonds with their bases [30]. Unlike the P and S motifs, however, the L motifs tend to have nonpolar amino acids at this position [13]. Therefore, it was proposed that the L motif functions as a spacer rather than an RNA base binder [12, 31]. Other groups have suggested that the L motif as well as the P and S motifs participates in RNA recognition [13, 14]. However, these hypotheses concerning the L motifs are based on computational analyses. Therefore, it was necessary to confirm experimentally whether L motifs contribute to base-specific RNA recognition.

To determine whether the L motif play a role in the RNA recognition, we first performed *in vitro* binding studies after expression in *E*. *coli* and purification of recombinant proteins. However, we were unable to purify full-length PpPPR_56 fused to a His-tag in C-terminus, although it was detected in the soluble fraction by immunoblot analysis using anti-His-tag antibody. The structure of the C-terminus of the DYW domain is yet unknown, but it is possible that the folding of the C-terminal region could hide the His-tag and prevent the purification of the full-length PpPPR_56 with Ni-NTA agarose beads. A recombinant PpPPR_56 lacking the DYW domain was designed to bypass this problem. However, it has been recently shown that the DYW domain of CRR2 influences the specific interaction between the E domain and its target RNA [9]. Therefore, we cannot exclude the possibility that the absence of the DYW domain in PpPPR_56 affected its binding affinity to target RNAs. The fusion of an epitope tag at the N-terminus may allow purification of the full-length PpPPR_56 protein.

Since the original PPR code was proposed [12, 14], bioinformatics [13, 32] and biomolecular studies [33] aimed to improve the PPR code. Yan *et al*. [33] tested the RNA binding affinity of 62 amino acid combinations using designer PPR proteins by REMSA. They have shown that [VN] and [VD] combinations found in 1L, 7L and 13L recognize adenine (A) and guanine (G), respectively, while [MD] and [LD] combinations found in 4L and 10L showed no binding to RNA. After studying the correlation between the amino acids and the aligned nucleotide, Kobayashi *et al* [32] proposed that the [VN], [MD], [VD], and [LD] combinations recognize preferentially A, U, U, and U, respectively. In PpPPR_56, only the 10L[LD] follows the proposed PPR code by recognizing U in *nad4*. Here, we showed that 56ΔDYW-nad3L, a PpPPR_56 variant with completely altered L motifs to recognize the *nad3* sequence, exhibited no significant difference from 56ΔDYW-wt in terms of *nad3* RNA binding activity (Figs 1 and 2), while modification of even a single P or S motif drastically change the binding specificity of PPR proteins [12, 16, 34]. This observation suggests that the original L motifs in PpPPR_56 do not mainly participate in RNA recognition *in vitro*, supporting the hypothesis that L motifs in PpPPR_56 work as spacers. This finding corroborates the conclusion that the alteration in one of the L motifs had no influence on the binding affinity of the *Arabidopsis* PLS-class protein CLB19 [16].

Although 56ΔDYW-nad3L could bind to both target RNAs *in vitro*, we failed to recover the RNA editing ability at *nad3*-C230 and *nad4*-C272 sites in *PpPPR_56* KO moss expressing *56nad3L* (Fig 3). This observation suggests that the original combination of amino acids in the L motifs of PpPPR_56 is irrelevant to RNA recognition but is essential for RNA editing. Mutations into the PPR motifs revealed that the involvement of the C-terminal L motifs in RNA editing (Figs 4 and 6). Polar amino acids at position 5 of the C-terminal L motifs also played a role in RNA editing (Fig 5). Thus, the C-terminal L motifs with nonpolar amino acids at position 5 are important for efficient editing. The last PLS triplet is different from the canonical PLS triplet in the amino acid frequency and systematic mutations of the amino acid residue at position 5 of the L2 motif (13L) impaired editing more than the last canonical L motif (10L) (Fig 5). Therefore, we cannot rule out that the recognition mechanism and/or the function of L2 motifs are different from the upstream L motif.

Based on the above results, we propose that the L motifs with non-polar amino acids at position 5 could act as non-binding spacers, rather than RNA binding motifs, to relax the structural constraints at least in the *P*. *patens*. This would allow formation of a complex between PLS-class PPR protein and RNA to correctly place the DYW domain in proximity to the editing site. Correct positioning between the DYW domain and the C to be edited is crucial for RNA editing. It was recently shown that the DYW domain of PpPPR_56 exhibits cytidine deaminase activity in *E*. *coli* [26]. The 56nad3L variant was unable to edit the target RNAs *in vivo* (Fig 3) possibly because polar amino acids at position 5 of the L motifs alter the structure of each PPR motif in PpPPR_56. This structural modification may cause a conformational change in the PPR-RNA complex and further interfere with the correct positioning of the DYW domain. The C-terminal L motifs are located near the DYW domain. Hence, RNA editing is severely impaired upon their mutations. In moss PPR proteins, P and S motifs also tolerate mismatches [18–22] suggesting that those motifs could be also important to keep the phase between the PPR motifs and the aligned RNA bases. This hypothesis might also explain the presence of more degenerated PPR motifs in the middle of the P-class PPR proteins and the difficulties experienced in the design of long rigid synthetic PPR proteins [5, 34].

The L motifs in the designer PLS-class protein without the DYW domain contribute to its interaction with one of the multiple organellar RNA editing factors (MORFs, also known as RNA editing factor interacting proteins [RIPs]) in order to increase the RNA binding activity of the PPR protein [31]. MORF/RIPs are absent in *P*. *patens*. Single *P*. *patens* DYW-type editing factors can edit their target RNAs in *E*. *coli* [26]. However, we cannot exclude the possibility that the modification of the *P*. *patens* L motifs may affect their interaction with other alternate editing factors and consequently impair RNA editing. Future study on the crystal structure of the PLS-class PPR protein with a C-terminal DYW domain may help elucidate the roles of L motifs.

PPR proteins are the potential candidates for RNA manipulation tools because each PPR motif recognizes one nucleotide, based on a simple code comprising two or three amino acids [35]. This mechanism resembles the mechanisms of transcription-activator-like effectors (TALEs) [36, 37] and Pumillio and FBF homology proteins (PUFs) [38]. DNA/RNA engineering tools based on TALEs or PUFs, such as artificial nucleases, have already been developed [39, 40]. In a recent study, Rojas *et al*. [41] successfully engineered a P-class PPR10 to activate the expression of chloroplast transgene. In the same way, PLS-class PPR proteins could be engineered and modified to edit any cytidine in any transcript. Here, we provide the experimental data highlighting the role of L motifs in RNA editing. To engineer PLS-class proteins, however, further refinement of the RNA recognition code of PPR proteins is needed.

## Supporting information

S1 Fig Recombinant PpPPR_56 and its variants showed no binding to the non-target *psbI*. (A) Schematic diagrams of the recombinant 56ΔDYW-wt and its variants (56ΔDYW-nad3L, 13L[PD], and 13L[ED]). Amino acids at positions 5 and last of each PPR motif are indicated. Altered amino acid residues are shown in red. Trx: thioredoxin; V5: V5 epitope tag; and His: 6× histidine tag. (B) Equal amounts (1 μg) of r56ΔDYW proteins (wt, nad3L, 13L[PD], and 13L[ED]) were analyzed by SDS-PAGE and Coomassie Brilliant Blue (CBB) staining. Black arrowhead indicates the predicted size of the 56ΔDYW proteins. (C) REMSA was performed with 56ΔDYW proteins (wt, nad3L, 13L[PD], and 13L[ED]), and ^32^P-labeled *psbI* RNA (PpPsbI-RNA1 [20]). The concentration of recombinant proteins was 100 nM. U indicates an unbound probe.
(EPS)

S2 Fig Effect of the mutations of the amino acids at positions 5 or last of the C-terminal L motifs in PpPPR_56 *in vivo*. (A) Alignment of PpPPR_56 and its variants to *nad3* and *nad4* RNAs. Key amino acids at positions 5 and last of each PPR motif are indicated. Altered amino acid residues are shown in red. Aligned nucleotides are shaded in gray to indicate matches to the proposed RNA recognition codes. (B) RNA editing levels in *PpPPR_56* KO mosses complemented with variants. The cDNA sequencing chromatograms and RNA editing efficiencies (%) are shown. Black arrowheads indicate editing sites.
(EPS)

S3 Fig *In vitro* RNA binding assay with the 13L[PD] and 13L[ED] variants of PpPPR_56. (A) REMSA was performed using 50 nM of 56ΔDYW proteins (wt, nad3L, 13L[PD], and 13L[ED]) and ^32^P-labeled RNAs (*nad3* and *nad4*). B and U indicate bound and unbound probe, respectively. (B) The histogram shows the percentage of probes (*nad3* and *nad4*) shifted with 50 nM of 56ΔDYW-wt and its variants (56ΔDYW-nad3L, 13L[PD], and 13L[ED]). Error bars indicate ± SD (*n* = 3).
(EPS)

S1 Raw Images Original gel images for Figs 1B, 2B, 3B and 6C, S1 and S3 Figs. (PDF)

S1 Table Oligonucleotide primers used in this study. (XLSX)
