# Supplementary figures and images for: The L motifs of two moss pentatricopeptide repeat proteins are involved in RNA editing but predominantly not in RNA recognition
Source: PLoS One. 2020 Apr 29;15(4):e0232366. doi: 10.1371/journal.pone.0232366 (PMC7190159; doi:10.1371/journal.pone.0232366)

Fig 1B

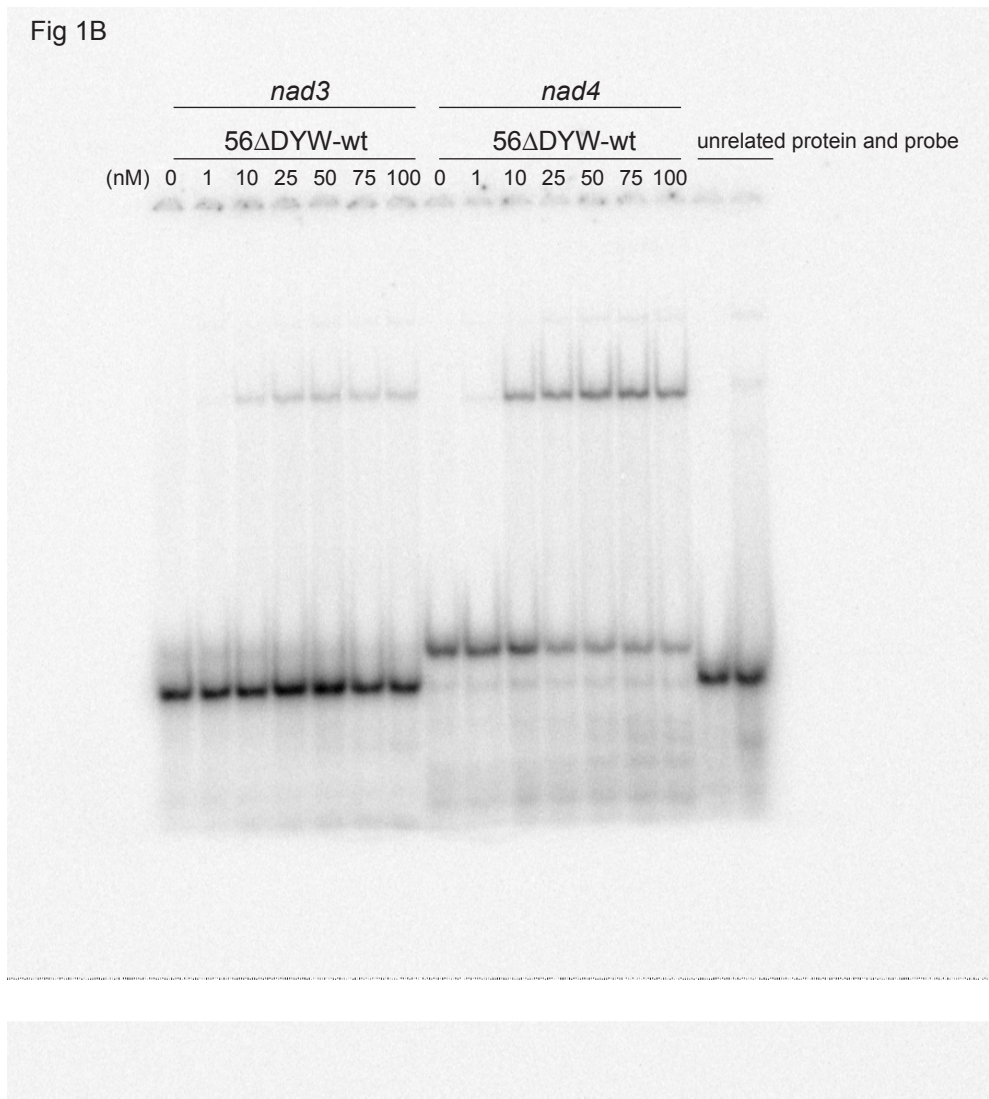

Fig 2B

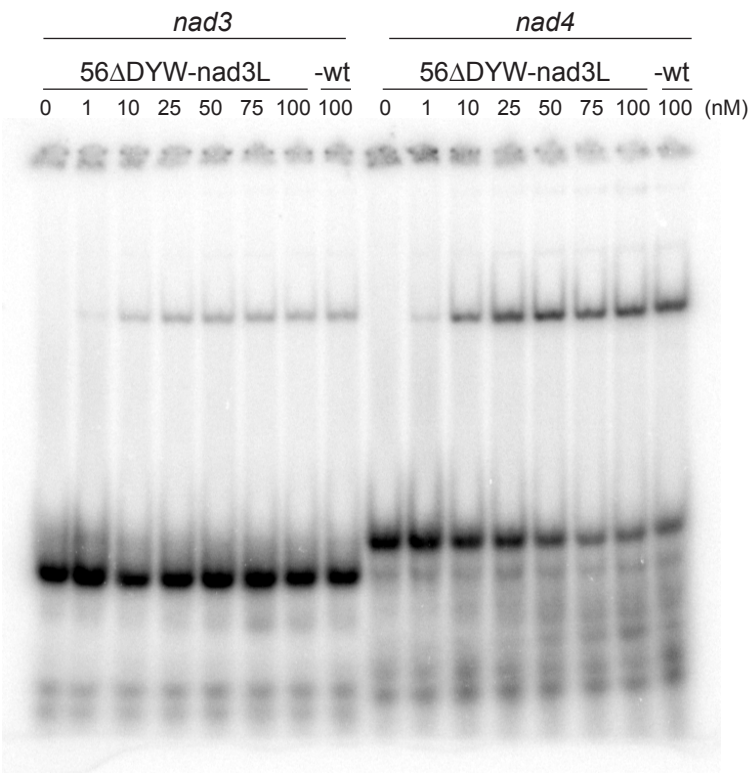

S1 Fig B

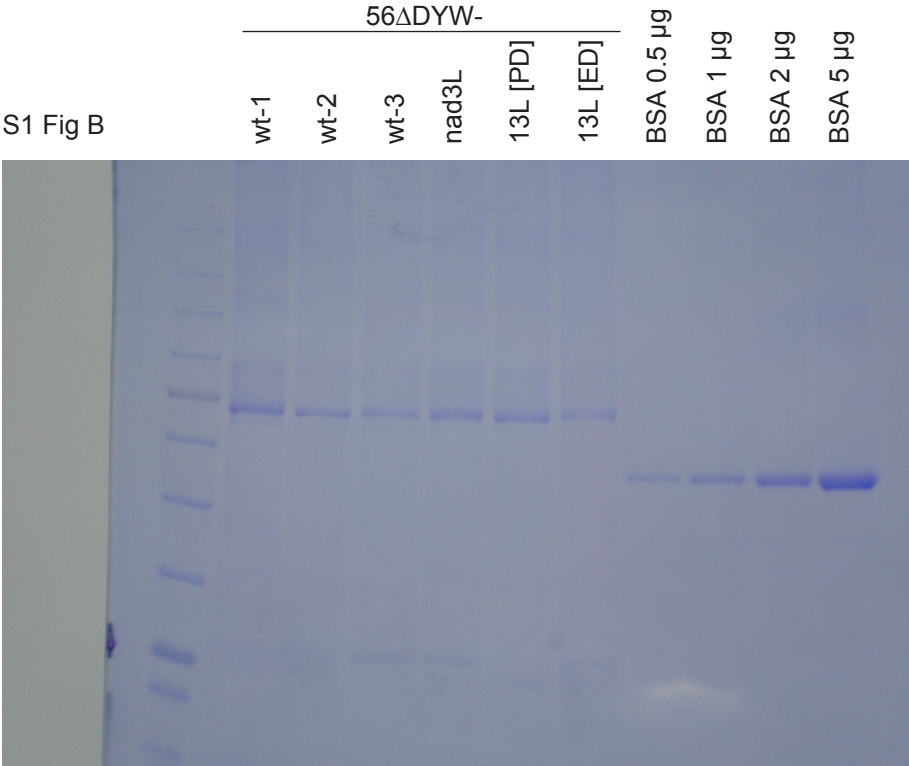

S3 Fig A

S1 Fig C

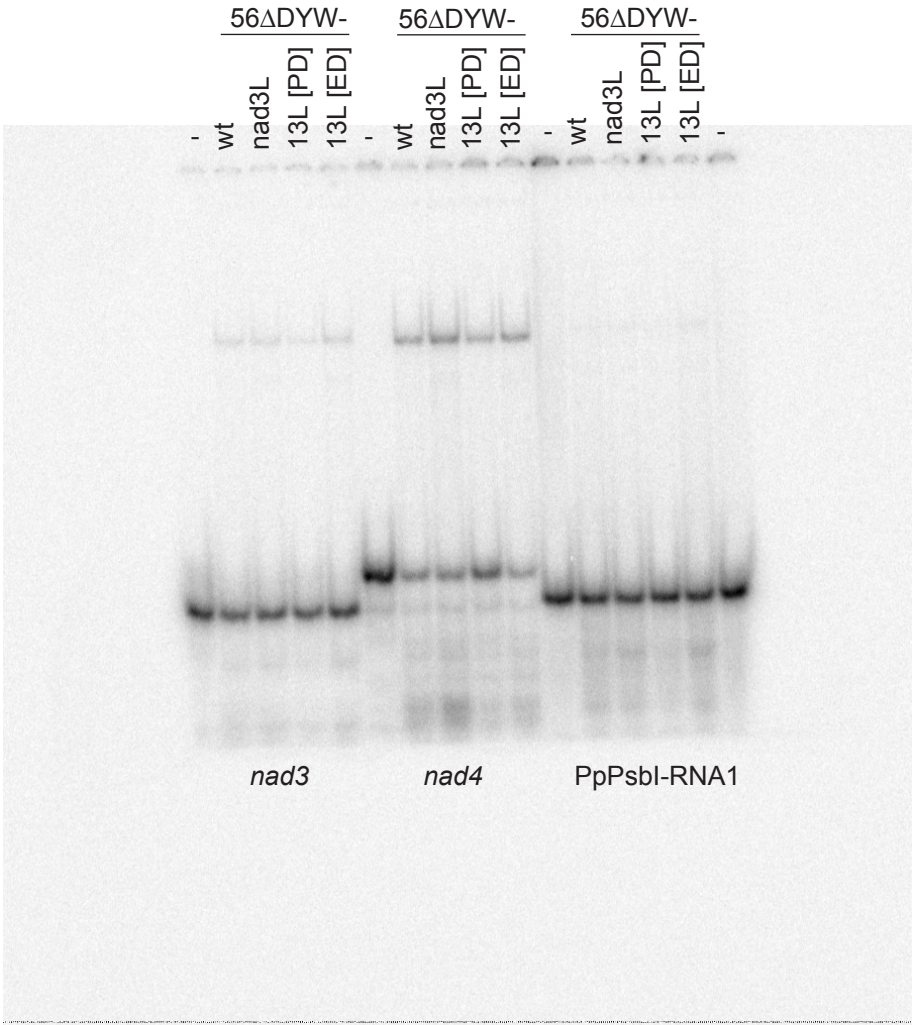

Fig 3B

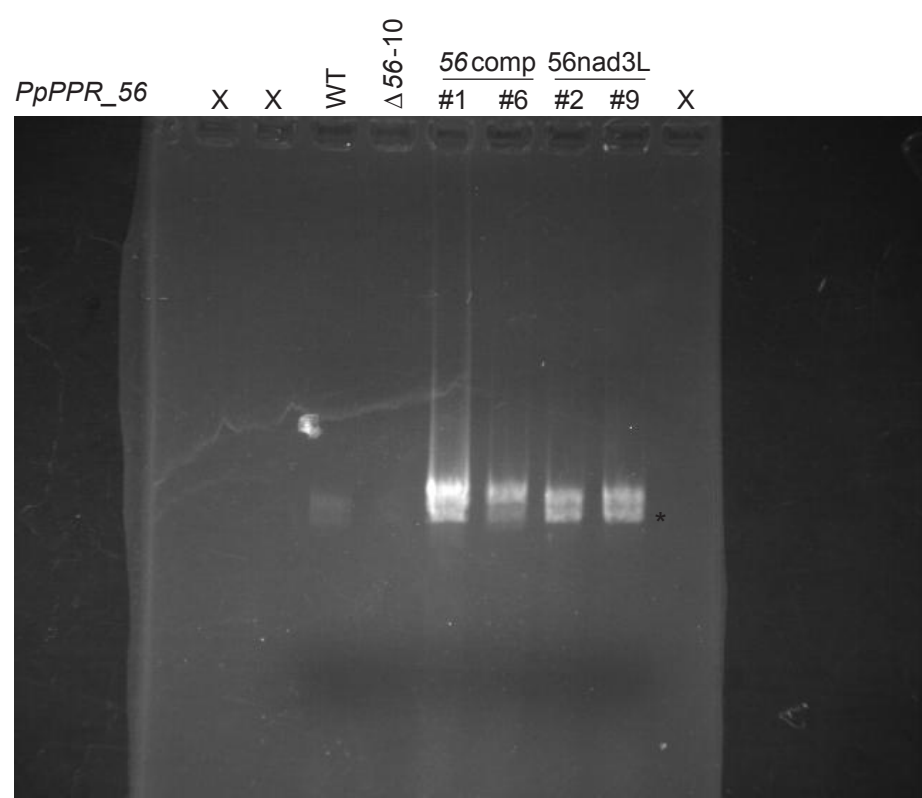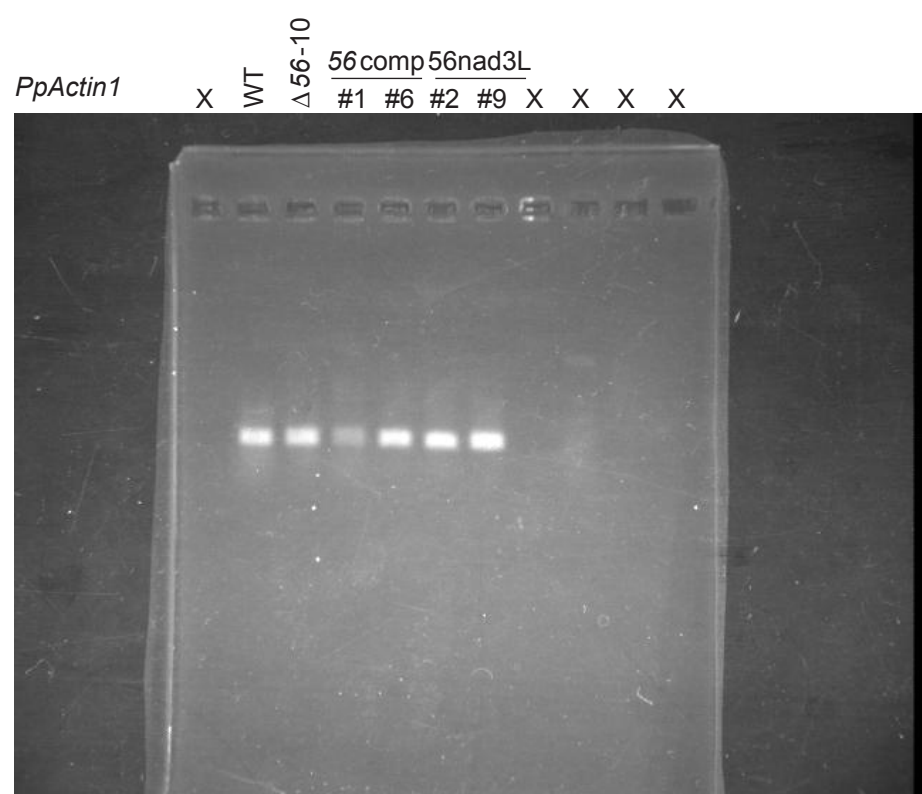

Fig 6C

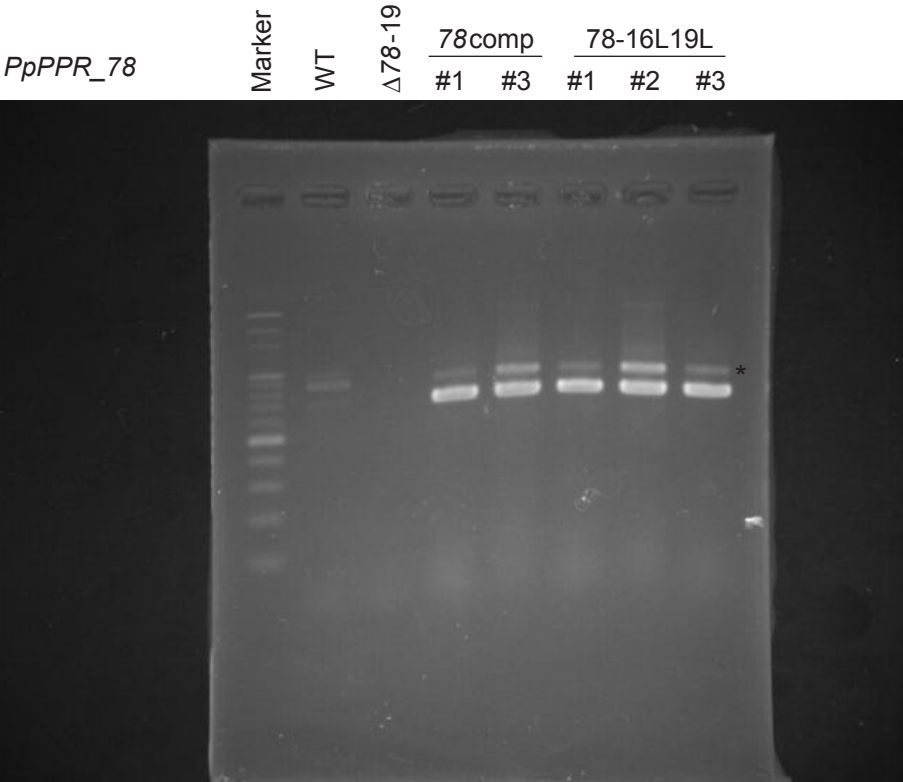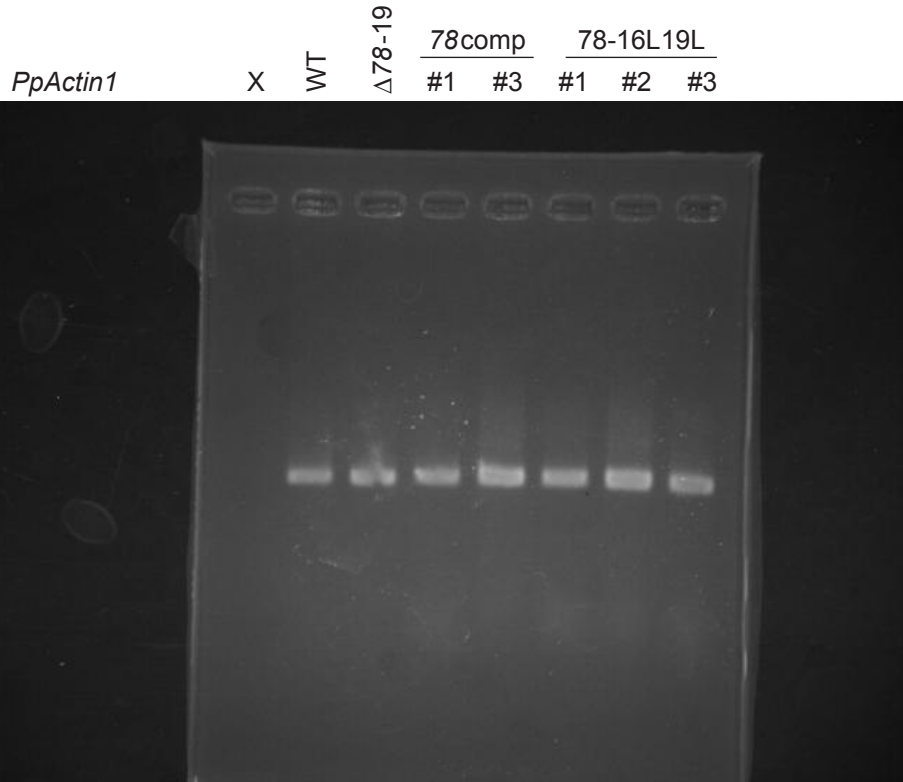

Supplement: S1 Raw Images — (PDF) [file pone.0232366.s004.pdf]
